# Predictive Analytics in Heart Failure Risk, Readmission, and Mortality Prediction: A Review

**DOI:** 10.7759/cureus.73876

**Published:** 2024-11-17

**Authors:** Qisthi A Hidayaturrohman, Eisuke Hanada

**Affiliations:** 1 Graduate School of Science and Engineering, Saga University, Saga, JPN; 2 Department of Electrical Engineering, Universitas Pembangunan Nasional Veteran Jakarta, Jakarta, IDN; 3 Faculty of Science and Engineering, Saga University, Saga, JPN

**Keywords:** heart failure, mortality, predictive analytics, predictive models, readmission, risk prediction

## Abstract

Heart failure is a leading cause of death among people worldwide. The cost of treatment can be prohibitive, and early prediction of heart failure would reduce treatment costs to patients and hospitals. Improved readmission prediction would also greatly help hospitals, allowing them to manage their treatment programs and budgets better. This literature review aims to summarize recent studies of predictive analytics models that have been constructed to predict heart failure risk, readmission, and mortality. Random forest, logistic regression, neural networks, and XGBoost were among the most common modeling techniques applied. Most selected studies leveraged structured electronic health record data, including demographics, clinical values, lifestyle, and comorbidities, with some incorporating unstructured clinical notes. Preprocessing through imputation and feature selection were frequently employed in building the predictive analytics models. The reviewed studies exhibit demonstrated promise for predictive analytics in improving early heart failure diagnosis, readmission risk stratification, and mortality prediction. This review study highlights rising research activities and the potential of predictive analytics, especially the implementation of machine learning, in advancing heart failure outcomes. Further rigorous, comprehensive syntheses and head-to-head benchmarking of predictive models are needed to derive robust evidence for clinical adoption.

## Introduction and background

Heart disease is one of the deadliest diseases among people worldwide [1], with 50% of heart failure (HF) patients dying within five years [2]. Doctors use a variety of tests to diagnose heart failure, including physical examination, blood and laboratory tests, and family history of the disease [1]. Even though there is no cure for heart failure, delicate medical procedures and treatments improve quality of life [2]. Researchers have conducted various studies to build prediction models to assist with the diagnosis of heart failure: early diagnosis allows a patient to get the proper treatment and minimizes the seriousness of this disease [3].

Medical procedures and treatments are expensive, especially for hospitalized patients [3]. To help reduce the expense, researchers have done numerous studies to identify heart failure patients who will need readmission. As information technology has developed, especially artificial intelligence, researchers have begun developing better early prediction systems. Including machine learning in the models improves their performance and prediction quality. With the right dataset and suitable data processing techniques, a predictive model’s performance should improve [4]. Predictive analytics has become the most used approach by researchers who construct predictive models using machine learning. In this approach, researchers use data gleaned from electronic health records to predict hospital readmission and mortality. However, there are serious obstacles to the development of predictive models that use a predictive analytics approach. Problems with data presentation and addressing problems such as imbalance in data class create challenges for researchers.

In this literature review, we provide an overview of predictive analytics methods, especially machine learning approaches, for predicting heart failure risk, readmission, and mortality among patients with heart failure. This narrative review aims to synthesize and interpret findings from a broad range of studies, providing a comprehensive overview of the machine learning methodologies employed in this domain. While traditional statistical approaches have been extensively used in clinical settings, our review highlights the advancements and contributions of machine learning techniques, which offer enhanced predictive capabilities by leveraging large, complex datasets. Through this narrative approach, we contextualize the role of machine learning in improving clinical decision-making and patient outcomes, offering insight into the potential and limitations of these technologies for heart failure risk prediction.

This article was previously posted to the Preprints.org preprint server on January 23, 2024, with doi https://doi.org/10.20944/preprints202401.1671.v1.

## Review

Methodology of literature selection

Literature Exploration

Before starting our search for research articles on predictive analytics in heart failure, it was necessary to define the targets, topics, and themes necessary for a comprehensive search. Five such targets were defined: predictive analytics in heart failure, heart failure risk prediction, heart failure readmission prediction, heart failure mortality prediction, and machine learning implementation in heart failure research. Furthermore, we chose closely related keywords that would be critical to our Google Scholar and PubMed search and limited it to the years from 2000 to 2023 to include only the most recent studies conducted in this field. Quotation mark- (“ ”) and Boolean operators (AND and OR) were used to search for titles and abstracts that were closely related to the defined topic. The final search query was: ("machine learning" AND ("heart failure" OR "heart failure prediction" OR "heart failure risk" OR "heart failure risk prediction")) AND ("predictive analytics" AND ("heart failure" OR "heart failure prediction" OR "heart failure risk" OR "heart failure risk prediction")). After doing this wide search, the next step was to select the studies to be included. The search stage resulted in 3,270 publications.

Study Selection and Screening

In the study selection step, several considerations went into pairing down the articles found in the preliminary search, as follows: 1. A paper must have been published in a journal or conference booklet, or book series to be selected; 2. It must be a research paper, not a review, a meta-analysis, or a literature review; 3. We considered the credibility and quality of the publisher. We cross-checked the publisher and journal with Scimago/Scopus and Clarivate/Web of Science to do this; 4. The published papers should include the full text. However, our university’s limited access to journal subscriptions may be problematic in terms of the selection of papers. Having access to the major publications but not all possible publications may have resulted in the exclusion of potentially relevant studies, thereby limiting the comprehensiveness of our review. Despite these constraints, we endeavored to mitigate this limitation by accessing open-access journals and utilizing interlibrary loan services whenever possible.

To eliminate papers that varied from the topic and to select those that met our conditions, we read the full titles and abstracts of the selected papers, paying careful attention to the considerations we set above, giving us 173 published papers. All were read with careful consideration given to the availability of data analysis and processing techniques, the use of machine learning or other approaches to build the predictive analytics model, and the data specification described in the articles. It was also important to ensure that the paper was related to the prediction of heart failure diagnosis, mortality, and readmission of heart failure patients. Papers meeting the above conditions in the screening stages were extracted for this review.

Data Extraction

Only 65 papers that met our specifications. The items extracted included the author’s name, year of publication, study objective, origin of data, dataset specifications, machine learning algorithm(s), methodology, and the evaluation of the model.

Literature Review Diagram Flow

**Figure 1:**
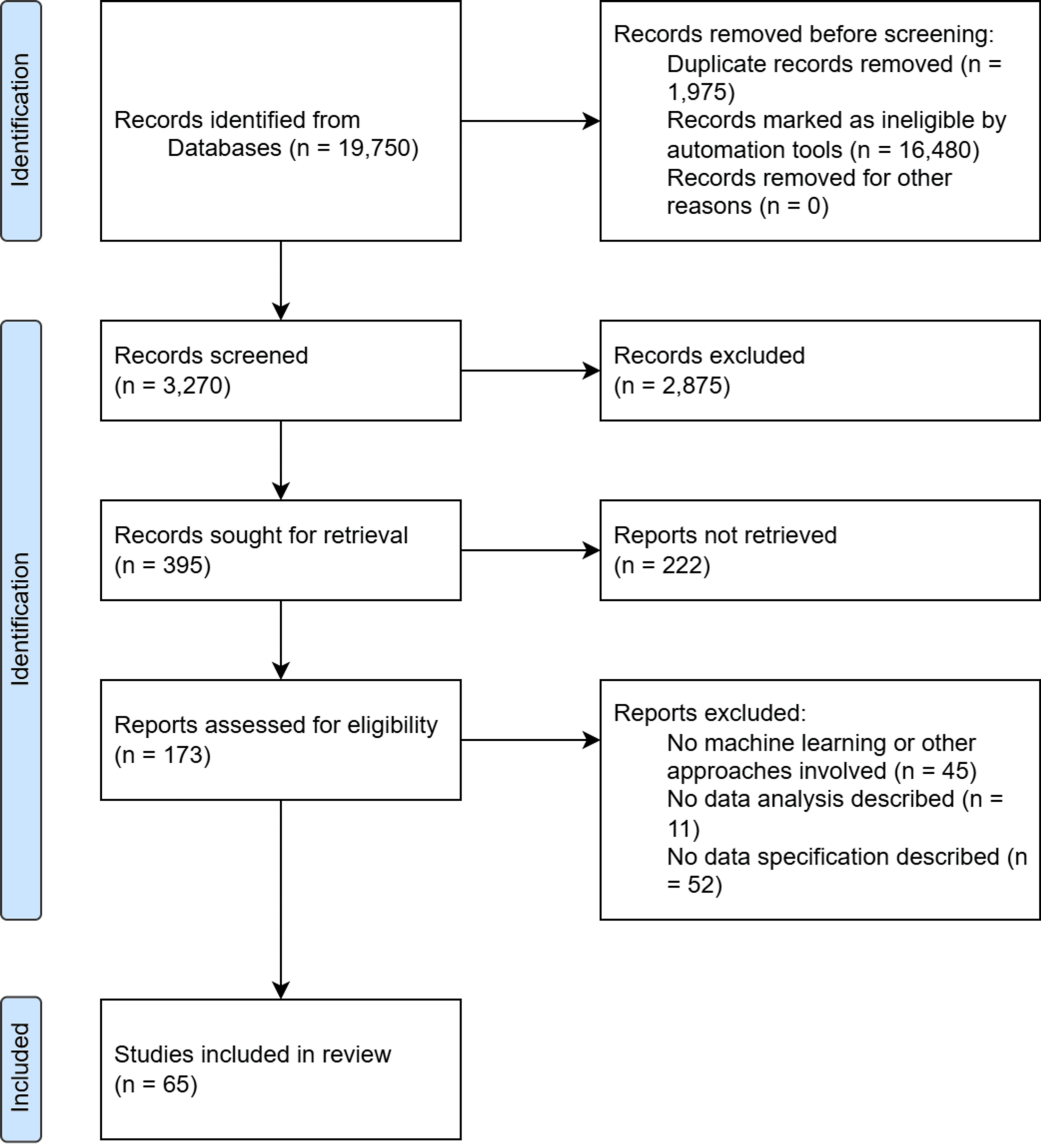
Literature review flow diagram

Figure 1 below shows the flow of this review process.

Classification of the Papers

The 65 papers extracted were classified into two categories. The first includes 26 papers that mainly aim to predict or diagnose heart failure or risk of heart failure using statistical or machine learning approaches to build a predictive analytics model for heart failure prediction, using either their own or open-accessed datasets. The second category includes 39 papers that aimed to predict readmission or mortality among patients with heart failure. They were divided into three subcategories: readmission (17), mortality (14), or both (8).

Results

Table 1 summarizes the studies on predictive analytics for heart failure risk, readmission, and mortality prediction. It includes diverse data sources like hospitals, clinics, and national databases, with sample sizes ranging from small cohorts to over a million patients. The studies focus on predicting heart failure diagnosis, readmission, and mortality using models such as random forest, neural networks, support vector machines (SVM), and logistic regression. Performance metrics like accuracy and AUC are used, with notable results including high accuracy and area under the curve (AUC) values, highlighting the potential of machine learning to enhance predictive analytics in heart failure.

**Table 1:** Summary of the included studies Abbreviations: NN:Neural Networks; SVM:Support Vector Machine; RF:Random Forest; DT:Decision Tree; CART:Classification and Regression Tree; kNN:k-Nearest Neighbor; LR:Logistic Regression; CNN:Convolutional Neural Network; LMT:Logistic Model Tree; ROT:Rotation Forest; XGBoost:eXtreme Gradient Boosting; Adaboost:Adaptive Boosting; LEBoosting:Least Error Boosting; LPBoosting:Linear Programming Boosting; NB:Naive Bayes; LightGBM:Light Gradient-Boosting Machine; MLP:Multilayer Perceptron; DNN:Deep Neural Networks; GB:Gradient Boosting; VFI:Voting Feature Intervals; GLMN:Generalized Linear Model Net; NLP:Natural Language Processing; LSTM:Long Short-Term Memory; SVC:Support Vector Classifier; BRF:Boosted Random Forest; ANN:Artificial Neural Networks; LASSO:Least Absolute Shrinkage and Selection Operator; PAR:Potentially Avoidable Readmissions; CMS:Centers for Medicare & Medicaid; PPR:Potentially Preventable Readmissions; SMOTE:Synthetic Minority Over-sampling Technique; KS-Test:Kolmogorov-Smirnov Test; PCA:Principal Component Analysis; RFE:Recursive Feature Elimination; AUC:Area Under the ROC curve; AUROC:Area Under Receiver Operating Characteristic Curve; PPV:Positive Predictive Values; NPV:Negative Predictive Values; SHAP:Shapley Additive Explanations; LR+:Positive Likelihood Ratio; NNT:Number Needed to Treat; PR-AUC:Precision Recall Aread under curve; MCC:Matthew's Correlation Coefficient; HF:Heart Failure; ECG:Electrocardiogram; BNP:Brain Natriuretic Peptide; KSUMC:King Saud University Medical City; NMMC:Northern Mindanao Medical Center; MARKER-HF:Machine Learning Assessment of Risk and Early Mortality in Heart Failure; UCI:University of California Irvine; UCSD:University of California San Diego; BIOSTAT-CHF:Biology Study to Tailored Treatment in Chronic Heart Failure; GWTG-HF:Get With The Guidelines-Heart Failure; ADHERE:Acute Decompensated Heart Failure National Registry; SHFM:Seattle Heart Failure Model; GISC:Gestione Integrata dello Scompenso Cardiaco

Study	Data Source	Sample Size	Prediction Target	Model(s) Used	Key Techniques	Performance Metrics	Result
Heart failure prediction studies that include diagnosis and risk prediction							
Guidi et al. [5]	St. Maria Nuova Hospital	136	HF severity	NN, SVM, Fuzzy genetic, CART, Random Forest	Developing CDSS for analysis HF patients	Accuracy	NN=84.73% SVM=85.2% Fuzzy genetic=85.9% CART=87.6% Random forest=85.6%
Yajuan et al. [6]	Geisinger Clinic	400,000+	HF diagnosis	Random Forest	Combining unstructured and structured data	AUC	Random forest=83%
Ng et al. [7]	Geisinger Clinic	400,000+	HF diagnosis	Logistic regression, Random Forest, SVM, kNN, Decision tree (Only L-1 logistic regression and Random forest were selected for superior predictive performance)	Processing longitudinal electronic health records data through feature extraction techniques	AUC	Both=0.74 - 0.80
Rammal [8]	KSUMC	100	HF diagnosis	Random Forest, Logistic regression	Big data environment and PCA	Accuracy, recall, precision, AUC	Random forest (%)=93.3, 93.3, 94.3, 94.2 Logistic regresstion (%)=93.3, 93.3, 93.3, 94.3
Nagrecha et al. [9]	Medicare USA	1 million+	HF diagnosis	Trajectory-based (Directed Acyclic Graph/DAG)	Disease progression	AUROC	DAG Depth: 1=0.5; 2=0.84; 3=0.82; 4=0.8
Krittayaphon et al. [10]	COOL-AF	3,461	HF risk factors	Cox Hazard proportional model	Calculating risk factors and incidence rate using Cox-proportional model	C-index, D-statistic, calibration plot, brier test, and survival analysis	C-index=0.756; D-statistic=1.503; R-square of the calibration plot=0.933; brier test=0.056
Austin et al. [11]	EFFECT Study	9,943	HFpEF prediction	Random Forest, Bagged decision tree, Boosted decision tree, SVM, Logistic regression	Comparison of predictive ability of different regression and classification methods	c-statistic, brier score, sensitivity, and specificity	classification tree=0.683, 0.2152, 0.462, 0.820; bagged tree=0.733, 0.2079, 0.451, 0.849; random forest=0.751, 0.1959, 0.378, 0.897; boosted tree (depth 1)=0.752, 0.2049, 0.453, 0.876; boosted tree (depth 2)=0.768, 0.1962, 0.491, 0.847; boosted tree (depth 3)=0.772, 0.1933, 0.492, 0.828; boosted tree (depth 4)=0.780, 0.1861, 0.500, 0.820; SVM=0.766, 0.1914, 0.401, 0.887
Blecker et al. [12]	Tisch Hospital	37,229	ADHF identification	Logistic regression, L1-regularization model	Comparing various approaches in identifying patients with ADHF	AUC, sensitivity, PPV	algorithm 1=n/a, 0.98, 0.14; algorithm 2=0.96, 0.98, 0.15; algorithm 3=0.99, 0.98, 0.30; algorithm 4=0.99, 0.98, 0.34
Plati et al. [13]	UCD and Ioannina Hospital	487	HF classification	Decision tree, random forest, rotation forest, naïve bayes, kNN, SVM, logistic model tree, and bayes network	Comparing five models with various sets of features including the implementation of pre-processing like balancing	Accuracy, sensitivity, specificity	Clinical features+LMT(%)=84.12, 82.10, 85.38; Clinidal features and BNP+LMT(%)=88.15, 85.80, 89.62; Clinical and ECG features+ROT(%)=90.76, 93.21, 89.23; ECG features+ROT(%)=87.91, 90.74, 86.15; All features+ROT(%)=91.23, 93.83, 89.62
Wang et al. [14]	Medical University Hospital in Shanxi Province	5,004	HF prediction	LR, kNN, SVM, RF, XGBoost	SHAP for model interpretation	AUC	LR=0.7819; kNN=0.6481; SVM=0.6963; RF=0.7983; XGBoost=0.8010
Quesada et al. [15]	ESCARVAL RISK	32,527	Cardiovascular risk	15 machine learning methods	Risk scale comparison	AUC, accuracy, error rate, sensitivity, specificity, PPV, NPV, LR+, and NNT	Top-3 best performed ML algorithm: QDA=0.7086, 0.570, 0.430, 0.736, 0.559, 0.0.093, 0.972, 1.669, 10.7; NB=0.7084, 0.591, 0.409, 0.718, 0.583, 0.096, 0.971, 1.722, 10.4; NN=0.7042, 0.559, 0.441, 0.743, 0.548, 0.092, 0.972, 1.644, 10.9
Kolukula et al. [16]	UCI Database	918	HF diagnosis	Logistic regression, SVM, random forest	Applying exploratory data analysis before model building	Accuracy, precision, recall, F1 score	LR=0.85, 0.82, 0.84, 0.845; SVM=0.84, 0.83, 0.83, 0.840; RF=0.986, 0.978, 0.981, 0.984
Sornsuwit et al. [17]	UCI Database	299	HF diagnosis	kNN, naïve bayes, decision tree, adaboost, bagging, logistic regression, LEBoosting	Developing a new classifier by modifying adaboost m1 classifier using naïve bayes, kNN, and decision tree	Accuracy	kNN=62.22; NB=68.89; decision tree=67.78; Adaboost=95.69; bagging=67.78; logistic regression=68.89; LPBoost=70; LEBoosting=98.89 (%)
Ahmed et al. [18]	UCI Database	500	HF diagnosis	Logistic regression, kNN, gaussian naïve bayes, multinomial naïve bayes, SVM	Combining public dataset with local dataset	Accuracy	Logistic regression (82.76%), SVM (67.24%), KNN (60.34%), GNB (79.51%), and MNB (79.51%).
Praveena et al. [19]	UCI Database	918	HF diagnosis	kNN, SVM, and random forest	Removing missing values	Sensitivity, precision, specificity, accuracy	kNN=0.7963, 0.7818, 0.7073, 0.75789; SVM=0.8, 0.8, 0.725, 0.76842; RF=0.8654, 0.8182, 0.7674, 0.82105
Alotaibi [20]	UCI Database	918	HF diagnosis	Decision tree, logistic regression, random forest, naïve bayes, SVM	Imputation implementation to public dataset	Accuracy	DT=93.19; LR=87.36; RF=89.14; NB=87.27; SVM=92.30 (%)
Mamun et al. [21]	UCI Database	299	HF diagnosis	LightGBM, XGBoost, Logistic regression, bagging, SVM, decision tree	SMOTE implementation of imbalance public dataset	Accuracy, AUC	LR(%)=80, 89.44; SVM(%)=73.22, 86.30; XGBoost(%)=84.00, 92.00; LightGBM(%)=85.00, 93.00; DT(%)=73.21, 73.20; bagging(%)=82.00, 89.05
Nishat et al. [22]	UCI Database	299	HF diagnosis	Decision tree, logistic regression, naïve bayes, random forest, kNN, SVM	SMOTE with normalization of imbalance public dataset	Accuracy, precision, F1 score, recall, AUC	DT=0.733, 0.756, 0.795, 0.838, 0.720; LR=0.800; 0.878, 0.857, 0.837, 0.755; GNB=0.683, 1.000, 0.812, 0.683, 0.500; RF=0.800, 0.854, 0.854, 0.854, 0.769; kNN=0.667, 0.902, 0.787, 0.698, 0.530; SVM=0.683, 1.000, 0.812, 0.683, 0.500
Senan et al. [23]	UCI Database	299	HF diagnosis	SVM, kNN, decision tree, random forest, logistic regression	SMOTE implementation of imbalance public dataset	Accuracy, precision, recall, F1 score	SVM(%)=90.00, 93.02, 93.02, 93.02; kNN(%)=93.33, 93.33, 97.67, 95.45; DT(%)=95.00, 93.48, 100, 96.63; RF(%)=95.00, 97.62, 05.35, 96.47; LR(%)=88.33, 93.00, 90.90, 91.93
Al-Yarimi et al. [24]	UCI Database	918	HF diagnosis	Decision tree, kNN, SVM	KS-Test for feature selection	Precision, accuracy, sensitivity, specificity, F-measure, Matthews correlation coefficient	FODW=0.89912, 0.911102, 0.954829, 0.872451, 0.88852, 0.823278; HRFLM=0.86574, 0.888546, 0.931869, 0.815411, 0.84294, 077306; HIFS=0.84712, 0.86347, 0.91806, 0.80536, 0.82544, 0.736158
Bharti et al. [25]	UCI Database	918	HF diagnosis	Logistic regression, kNN, SVM, random forest, decision tree, deep learning	Lasso for feature selection	Accuracy, specificity, sensitivity	LR(%)=83.3, 82.3, 86.3; kNN(%)=84.8, 77.7, 85.0; SVM(%)=83.2, 78.7, 78.2; RF(%)=80.3, 78.7, 78.2; DT(%)=82.3, 78.9, 78.5; Deep Learning(%)=94.2, 83.1, 82.3
Kanagarathinam et al. [26]	UCI Database	918	HF diagnosis	Naïve bayes, XGBoost, kNN, SVM, MLP, CatBoost	Pearson's correlation for feature selection	Accuracy, AUC	Accuracy only (%) Naïve Bayes=85.98; XGBoost=86.91; kNN=85.98; SVM=85.98; MLP=85.98; CatBoost=87.85
Venkatesh et al. [27]	UCI Database	918	HF diagnosis	Naïve bayes	Big data environment	Precision, recall, F1-score	NB=0.80, 0.82, 0.81
Alsubai et al. [28]	UCI Database	918	HF diagnosis	Decision tree, SVM, random forest	Quantum computing	Accuracy, precision, recall, F1 score, AUC	DT=0.62, 0.65, 0.63, 0.61, 0.633; SVM=0.70, 0.71, 0.70, 0.70, 0.699; RF=0.79, 0.79, 0.79, 0.79, 0.787; QDL=0.98, 0.98, 0.98, 0.98, 0.98
Botros et al. [29]	MIT-BIH and BIDMC	18	HF diagnosis	CNN, CNN-SVM	ECG signal analysis	Accuracy, sensitivity, specificity	CNN(%)=99.31, 99.50, 99.11; CNN-SVM(%)=99.17, 99.74, 98.61
Alsinglawi et al. [30]	MIMIC-III	1,592	HF patient's LOS prediction	Random forest, gradient boosting, stacking regression, DNN	Predicting length of stay using EHR-based dataset	R-squared, mean average error	RF=0.8, 1.98; GB=0.81, 2.0; stacking regression=0.81, 1.92; DNN=0.77, 2.30
Readmission Prediction							
Shameer et al. [31]	Mouth Sinai Hospital	1,068	Hospital readmission	Naïve bayes	Applying five different data modalities based on feature's type	Accuracy and AUC score	NB=83.9% and 0.780
Bat-Erdene et al. [32]	KAMIR-NIH registry	13,104	Rehospitalization prediction	Logistic regression, SVM, gradient boosting, AdaBoost, random forest, proposed DNN	Modifying deep neural network and comparing with other ML-based models	Accuracy, AUC, precision, recall, specificity, F1-score	LR(%)=94.37, 95.82, 88.51, 75.82, 97.30, 78.51; SVM(%)=98.53, 99.60, 99.32, 89.88, 99.90, 94.27; gradient boosting(%)=97.58, 98.73, 94.94, 86.91, 99.27, 90.70; AdaBoost(%)=95.89, 97.73, 86.01, 83.21, 97.88, 84.56; RF(%)=97.98, 98.75, 99.41, 85.67, 99.91, 91.98; DNN(%)=99.37, 99.90, 96.86, 98.61, 99.49, 97.73
Rizinde et al. [33]	Seven hospitals in Rwanda	4,083	Risk of hospitalization prediction	Random forest, SVM, kNN, MLP, logistic regression, decision tree	Applying various pre-processing techniques including balancing and comparing with various models	Accuracy, precision, recall, F1 score, AUC	RF(%)=87, 84, 89, 87, 94; SVM_RBF(%)=79, 77, 82, 79, 88; SVM_Linear(%)=74, 73, 76, 74, 88; kNN(%)=85, 80, 91, 85, 88; MLP(%)=82, 79, 86, 82, 88; LR(%)=75, 74, 75, 74, 81; DT(%)=52, 50, 96, 66, 57
Landicho et al. [34]	NMMC	322	Readmission of HF patients	Logistic regression, SVM, random forest, neural network	Applying cost-senstive classification approach to the model	Accuracy, sensitivity, specificity, precision, F-measure, AUC	LR=0.585, 0.250, 0.904, 0.714, 0.370, 0.577; RF=0.561, 0.150, 0.952, 0.750, 0.250, 0.551; SVM=0.610, 0.300, 0.905, 0.750, 0.428, 0.602; NN=0.512, 0.000, 1.000, -, -, 0.500
Sohrabi et al. [35]	Iran Medical Centers	230	Re-hospitalization of HF patients	Decision tree, artificial neural networks, SVM, logistic regression	Data mining approach for data analytics	Accuracy, AUC	Re-hospitalization 1 month DT=88.1%, 0.76; ANN=86.5%, 0.68; SVM=86.4%, 0.62; LR=80.1%, 0.61
AbdelRahman et al. [36]	University of Utah Health Care	2,787	Readmission of CHF patients	Voting feature intervals classifier, logistic regression, combination VFI and logistic regression	Utilizing three-step approaches	AUC, accuracy, sensitivitiy, specificity, PPV, NPV	Voting classifier(%)=86.8, 91.5, 62.5, 94.2, 50, 96.4
Vedomske et al. [37]	University of Virginia CDR	1 million+	30-day readmission of CHF patients	Random forest	Processing administrative data	AUC	RF-based models with prior weighting on the response based on divided variables: Base=0.67; Procedure=0.68; Diagnosis=0.77; Both=0.8
Hilbert et al. [38]	California State Inpatient Database of the Healthcare Cost and Utilization Project	78,091	Readmission of HF patients	Decision tree	Transparant analysis of decision tree models	AUC	Training=0.594; Testing=0.583
Zolbanin and Delen [39]	CHSI at OSU	32,350	Readmission of HF patients	Naïve bayes, neural network, gradient boosting	Conducting database-wide data processing in model building	AUC	Naïve Bayes=0.733; neural network=0.728; gradient boosting= 0.722
Golas et al. [40]	Partners Healthcare System	28,031	30-day hospital readmission of HF patients	Modifiend deep learning, logistic regression, gradient boosting, neural network	Designing deep unified networks (DUNs) to avoid overfitting in predictive model	AUC, accuracy, precision, recall, f1	LR=0.664, 0.626, 0.336, 0.616, 0.435; gradient boosting=0.650, 0.612, 0.325, 0.615, 0.425; maxout networks=0.695, 0.645, 0.354, 0.631, 0.454; DUN=0.705, 0.646, 0.360, 0.652, 0.464
Mortazavi et al. [41]	Tele-HF	1,653	30-day hospital readmission of HF patients	Random forest, boosting, SVM, logisitc regression	Involving telemedicine-based data	Discrimination c-statistic	180 days heart failure readmission: LR=0.566; boosting=0.678; RF=0.669; RF into SVM=0.657
Lorenzoni et al. [42]	GISC	380	Hospitalization of HF patients	GLMN, logistic regression, CART, random forest, adaboost, logitboost, SVM, neural network	Involving three different approaches (complete case, kNN imputed, median imputed) in handling missingness	Sensitivity, PPV, NPV, specificity, accuracy, AUC	Only best performed algorithm, GLMN (%): Complete Case: 77.8, 87.5, 75, 85.7, 81.2, 80.6; Mean-imputation: 26.5, 66.0, 59.5, 68.1, 60.3, 62.8; kNN-imputation: 24.1, 64.8, 59.4, 89.5, 60.3, 62.4
Sundararaman et al. [43]	MIMIC-III	11,318	Readmission of HF patients	Logistic regression	Employing four model approaches involving structured and unstructured data	Accuracy and AUC score	structured data=0.91, 0.68; unstructured data=0.92, 0.63; feature selection=0.98, 0.96; structured + unstructured data=0.92, 0.64; structured + feature selection=0.98, 0.97
Liu et al. [44]	MIMIC-III	58,000+	Readmission of HF patients	NLP CNN	Employing only clinical notes	Accuracy	General readmission: CNN=0.759, 0. 754, 0.756, 75.70%; RF=0.720, 0.633, 0.674, 69.35%
Sharma et al. [45]	Alberta Health Services	10,641	Readmission of HF patients	12 ML models	Comparing 12 ML-based with LaCE score	AUC	Validation set: XGBoost=0.654; GBM=0.650; AdaBoost=0.646; CatBoost=0.642; LightGBM=0.641; Linear SVC=0.639; NB=0.624; RF=0.617; DT=0.597; LR=0.596; NN=0.578; LSTM=0.624; LaCE=0.570
Shams et al. [46]	Veteran Health Administration	7,200	Readmission of HF patients	PAR	Proposing PAR approach and comparing with CMS and PPR	AUC-ROC	PAR=0.836
Ben-Assuli et al. [47]	Sheba Medical Center	10,763	30-day readmission of HF patients	XGBoost	Involving expert cardiologists for feature selection	AUC	Machine superlist=0.8156; Expert list=0.7116; Human-Machine collaboration=0.8289
Mortality Prediction							
Jing et al. [48]	Geisinger EHR	270,000	Mortality	Logistic regression, random forest, XGBoost	Performing split-by-year training scheme and simulating care gap closure to the predictive models	AUC	Logistic regression=0.74; Random forest= 0.76, XGBoost=0.77
Kamio et al. [49]	Tokushukai Medical Database	1,416	In-hospital mortality for ICU-admitted patients with AHF	SVM, XGBoost, neural network	Proposing three types of data: static, time-seris, and a combination	F1-score, precision, recall, ROC AUC, PR AUC	Time-series & static data: LSVC=0.49, 0.38, 0.69, 0.74, 0.47; XGB=0.49, 0.42, 0.64, 0.73, 0.48; Multi-neural network=0.37, 0.41, 0.52, 0.65, 0.37
Adler et al. [50]	UCSD	5,822	Mortality risk	Boosted decision tree	Performing MARKER-HF score	AUC	Results for each cohort study: UCSD (all variables)=0.88; UCSD (RDW imputed)=0.87; UCSF=0.81; BIOSTAT-CHF=0.84
Lagu et al. [51]	HealthFacts data of Cerner Corporation	13,163	Mortality of patients with ADHF	Logistic regression	Comparing seven mortality prediction models	AUC	Results for each model: Premier + =0.81; LAPS2=0.80; Premier=0.76; EFFECT=0.70; GWTG-HF-Eapen=0.70; GWTG-HF-Peterson=0.69; ADHERE=0.68
Panahiazar et al. [52]	Mayo Clinic	119,749	Mortality	Decision tree, random forest, Adaboost, SVM, logistic regression	Comparing SHFM with ML-based models	AUC	Results of 1-year mortality prediction: SHFM-based: DT=0.60; RF=0.62; AdaBoost=0.59; SVM=0.56; LR=0.68 Proposed model: DT=0.66; RF=0.80; AdaBoost=0.74; SVM=0.46; LR=0.81
Almazroi et al. [53]	UCI Heart Failure	299	Mortality	Decision tree, SVM, ANN, LR	Comparing three ML-based model to predict death event of HF patients	Accuracy, precision, recall, F1-score	LR(%)=78.34, 91.67, 47.82, 62.8; DT(%)=80, 78.94, 65.21, 71.4; SVM(%)=66.67, 80, 17.39, 28.57; ANN(%)=60, 40, 8.69, 14.28
Karakuş et al. [54]	UCI Heart Failure	299	Mortality	Logistic regression, naïve bayes, SVM, kNN, decision tree, random forest, neural network	Utilizing PCA to determine the effectiveness in prediction performance	Accuracy, AUC	Accuracy was achieved between 69 and 100% AUC: Gaussian SVM (1.00) and Coarse kNN (0.87)
Zaman et al. [55]	UCI Heart Failure	299	Mortality	Decision tree, random forest, XGBoost	Implementing SMOTE for balancing and two learners: base and meta	Accuracy, precision, recall, F1-score, AUC	DT(%)=80.49, 16.19, 84.21, 80.00; RF(%)=92.68, 88.09, 97.36, 92.50; XGBoost(%)=91.46, 87.80, 94.73, 91.13
Newaz et al. [56]	UCI Heart Failure	299	Mortality	SVM, kNN, logistic regression, adaboost, random forest	Presenting a robust BRF for imbalance problems	Senstivity, specificity, G-mean, accuracy, MCC, AUC	BRF + RFE(%)=78.21, 70.51, 74.26, 72.93, 46.33, 74.36; BRF + Chi2(%)=80.21, 74.45, 76.83, 76.25, 52.53, 77.33
Kedia et al. [57]	UCI Heart Failure	299	Mortality	Logistic regression, naïve bayes, decision tree, random forest, SVM	Performing feature selection RFE with SMOTE for balancing	Accuracy, precision, recall, F1-score	LR=85.56%, 0.82, 0.81, 0.81; GNB=85.56%, 0.83, 0.78, 0.80; DT=80.0%, 0.75, 0.77, 0.76; RF=88.89%, 0.85, 0.84, 0.84; Linear SVM=87.78%, 0.85, 0.84, 0.84; Stack model=90.00%, 0.88, 0.87, 0.87
Chicco et al. [58]	UCI Heart Failure	299	Mortality	Random forest, decision tree, gradient boosting, linear regression, ANN, naïve bayes, SVM, kNN	Combining ML- and biostatistical-based feature selection	Accuracy, F1-score, MCC, PR-AUC, ROC-AUC	Results of model after feature selection: RF=0.585, 0.754, +0.418, 0.541, 0.698; gradient boosting=0.585, 0.750, +0.414, 0.673, 0.792; SVM radial=0.543, 0.720, +0.348, 0.494, 0.667
Li et al. [59]	MIMIC-III	1,177	Mortality of ICU-admitted HF patients	XGBoost, LASSO, logistic regression	Utilizing ML-based model for screning independent risk factors for in-hospital mortality	AUC and calibration c-statistic test	AUC score of each model: XGBoost=0.8416; LASSO=0.8562; GWTG-HF=0.7747
Luo et al. [60]	MIMIC-III	5,676	Mortality of ICU-admitted HF patients	XGBoost, logistic regression	Utilizing imputation and ML-based feature selection	AUC, calibration plot, decision curve analysis	AUC score of each model: XGBoost=0.831; ElasticNet=0.817; SAPS-II=0.719; GWTG-HF=0.662
Chen et al. [61]	MIMIC-IV and eICU	20,878 and 15,483	Mortality of ICU-admitted HF patients	Xgboost, logistic regression	Utilizing an updated of MIMIC dataset and testing the proposed model with eICU dataset	AUC	XGBoost=0.771; Logistic=0.725; GWTG-HF=0.649
Readmission and Mortality Prediction							
Awan et al. [62]	Hospital Morbidity Data Collection and Mortality Database	248,387	Readmission and Mortality	logistic regression, random forest, decision tree, SVM, MLP	Comparing proposed models with LaCE score	AUC, PR-AUC, accuracy, sensitivity, specificity	LR=0.576, 0.455, 62.26%, 48.37%, 66.85%; Weighted-RF=0.548, 0.386, 76.22%, 21.71%, 88.07%; Weighted-DT=0.528, 0.379, 64.18%, 31.44%, 74.16%; Weighted-SVM=0.535, 0.377, 65.36%, 31.39%, 75.78%; MLP=0.628, 0.461, 64.93%, 48.42%, 70.01%
Awan et al. [63]	Hospital Morbidity Data Collection and Mortality Database	248,387	Readmission and Mortality	MLP	Leveraging feature selection and dimension reduction	sensitivitiy, specificity, AUC	Based on feature selection methods: forward selection=32.2%, 85.3%, 0.56; backward selection=44.2%, 66.6%, 0.57; mRMR=58.7% 60.6%, 0.62
Sarijaloo et al. [64]	EPIC EHR and the McKesson Change ECG Reporting System	3,189	90-day AHF readmission and mortality	SVM, random forest, gradient boosting, LASSO, logistic regression	Comparing ML-based models with traditional statistical analysis for risk assessment	AUC and 95% CI	LR=0.744, (0.732-0.755); ML-LASSO=0.748, (0.745-0.751); ML-GBM=0.750, (0.743-0.756); ML-RF=0.570, (0.545-0.594); ML-SVM=0.718, (0.703-0.733); Combined ML-LASSO+LR=0.760, (0.752-0.767)
Tian et al. [65]	Shanxi Province	1,011	Death, readmission, and MACEs	Logistic regression, random forest, XGBoost, LightGBM, naïve bayes, MLP	Developing chronic heart failure measure (CHF-PROM)	AUC	Final models for HF-readmission: XGBoost=0.718; LightGBM=0.704; RF=0.707; Logistic=0.693; NB=0.673; MLP=0.690 Final models for all-cause death: XGBoost=0.754; LightGBM=0.733; RF=0.709; Logistic=0.742; NB=0.658; MLP=0.746
Lv et al. [66]	Hospital of Dalian Medical University	13,602	1-year in hospital mortality and 1 year readmission	Logistic regression, SVM, ANN, random forest, XGBoost	Presenting calibration plots of each predictive models for comparison	calibration plot, AUC	All-cause mortality: LR=0.91; RF=1.00; SVM=0.99; ANN=0.99; XGBoost=0.99 All-cause readmission: LR=0.63; RF=0.91; SVM=0.96; ANN=0.82; XGBoost=0.92
Eapen et al. [67]	GWTH-HF	33,349	Readmission and Mortality	CMS tool	Demonstrating the use of CMS tool	C-statistics	Validation cohort: 30-day mortality=0.75; 30-day hospitalization=0.59; 30-day mortality or rehospitalization=0.62
Zhao et al. [68]	TOPCAT trial dataset	3,445	Readmission and Mortality of HFmrHF patients	Random forest, logistic regression, LASSO, RIDGE, gradient boosting, SVM	Presenting DeLong test to assess discrimination and its improvement	AUC, c-index	Death 1-year: RF=0.68, 0.67; LASSO=0.75, 0.77; Logistic=0.46, 0.53; Ridge=0.52, 0.51; GBT=0.77, 0.76; SVM=0.62, 0.64 HF-hospitalization 1-year: RF=0.84, 0.85; LASSO=0.47, 0.49; Logistic=0.58, 0.43; Ridge=0.39, 0.63; GBT=0.81, 0.81; SVM=0.40, 0.70
Beecy et al. [69]	CLEVER-HEART	3,774	30-day unplanned readmission and mortality	XGBoost	Comparing proposed models with HOSPITAL score	AUC	30-day outcomes: Index Admission=0.723; Index Discharge=0.754; Feature Aggregated=0.756; HOSPITAL score=0.666; Index Admission BNP=0.5046

Predictive Analytics for Heart Failure Prediction

The selected studies highlighted the important role of machine learning in predicting HF from electronic medical records. This approach may greatly help the clinical decision-making process and diagnose patients with heart failure. Guidi et al. designed a clinical decision support system (CDSS) by implementing machine learning in decision-making to evaluate the severity of HF among patients with HF [5]. The study underscored the readability and accessibility of machine learning-based CDSS to non-cardiologist users.

Involving more than 400,000 primary care patients, two studies built machine learning-based predictive analytics models for the early diagnosis of heart failure in primary care patients collected by the Geisinger Clinic [6,7]. By using unstructured and structured electronic health record (EHR) data, these studies were able to predict heart failure in different time windows and showed good performance. Similarly, Ramal and Emam utilized the data of 100 patients from King Saud University Medical City (KSUMC) in a big data environment [8]. Involving principal component analysis (PCA)’s pre-processing and feature reduction techniques, the study obtained a promising result in predicting heart failure

Aside from machine learning in building its predictive models, Nagrecha et al. involved more than one million elderly patients from Medicare USA and built predictive models using a trajectory-based disease progression model to predict heart failure among unseen patients [9]. Another study used the Cox Hazard proportional model to predict heart failure risk factors by patients collected by COOL-AF Thailand [10]. The proposed study performed well in predicting heart failure by calculating the model’s C-index, D-statistics, calibration plot, brier test, and survival analysis.

Austin et al. described the use of a machine learning-based predictive analytics approach to predict Heart Failure with preserved Ejection Fraction (HFpEF), a subtype of heart failure [11]. The study assessed the proposed models using c-statistic, brier score, sensitivity, and specificity and accurately predicted the presence of HFpEF in heart failure patients. Similarly, Blecker et al. used machine learning-based predictive analytics models to identify acute decompensated heart failure (ADHF) in patients collected by Tisch Hospital, USA [12]. The proposed study found that a machine learning-based predictive model best predicted ADHF.

Plati et al. involved 487 patients provided by the University College of Dublin (UCD) and the Department of Cardiology of the Hospital University Ioannina to build machine learning-based predictive analytics to classify the type of heart failure [13]. By performing pre-processing techniques and class balancing to obtain an ideal dataset, the study generated promising results for classifying heart failure by dividing the main dataset into a sub-dataset for each type.

Furthermore, Wang et al. underscored the interpretability of predictive analytics models [14]. The study described the use of model interpretation and feature importance explanation using the Shapley additive explanation (SHAP) approach to give physicians an understanding of the models. The study used the SHAP approach for the best-performed model to interpret the model and its feature importance. Additionally, comparing machine learning-based with commonly used risk scales showed the effectiveness of their predictive analytics approach [15]. The study showed that machine learning-based predictive analytics outperformed other risk scales like SCORE and REGICOR.

While various previously discussed studies rely on hospital data, there are selected studies that build predictive models that leverage open, public data from repositories such as Physionet and University of California Irvine (UCI). Three selected studies used heart disease datasets from the UCI machine learning database to build predictive models for diagnosing heart failure or heart disease [16-18]. By directly processing the dataset, the studies employed various machine learning algorithms. However, challenges arose due to missing values that existed in the dataset. Removing all the missing values was shown to be fundamental to obtaining a dataset without missing values [19]. However, this might result in biased prediction results. By implementing an imputation technique, Alotaibi showed better performance [20].

In other cases, an imbalance problem may have caused a reduction in prediction performance. Mamun et al. showed that the SMOTE technique successfully addresses the imbalance in the UCI heart disease dataset, and it improved the predictive performance [21]. However, the number of UCI heart disease patients differed slightly in each class, so this technique was unnecessary. Nishat et al. and Senan et al. utilized the SMOTE technique to solve the imbalance problem in the UCI heart failure dataset [22,23]. Furthermore, by implementing normalization techniques, Nishat et al. showed improved prediction performance compared to models without balancing and normalization, as Senan et al. did.

Selecting important features may lead to predictive performance improvement. Leveraging datasets from the UCI database, Al-Yarimi et al. adopted the KS-Test to select the optimal attributes for their dataset and build a decision tree-based predictive model [24]. However, according to Mathew’s correlation test to evaluate the predictive model, the model's performance was not significantly improved. Bharti et al. used Lasso for feature selection, resulting in better classification performance than was seen with the KS-Test approach [25]. Differing from those studies, Kanagarathinam et al. performed feature selection using Pearson’s correlation to obtain an ideal version of the UCI heart disease dataset [26]. This study shows improved predictive performance compared to previously mentioned studies. However, the result might be biased because the study discarded variables with missing values.

Unlike other studies, Venkatesh et al. used a big data approach and the UCI heart disease dataset to analyze and predict heart status [27]. The study improved the prediction effectiveness with high CPU utilization and low processing time by utilizing a clustering technique to filter unnecessary data. Alsubai et al. implemented quantum computing in machine learning and deep learning algorithms, resulting in better predictive performance than conventional machine learning algorithms [28].

Utilizing datasets from Physionet, Botros et al. used datasets from the MIT-BIH and BIDMC databases to predict heart failure by analysis of ECG signals [29]. By leveraging a deep learning algorithm, the study was able to build a predictive model with good performance in terms of accuracy, sensitivity, and specificity. Using the MIMIC-III dataset, Alsinglawi et al. were able to predict the length of stay for patients with heart failure using machine learning-based predictive analytics [30].

The selected studies underscore the benefit of implementing predictive analytics in heart failure prediction, offering valuable insights for clinicians and physicians.

Predictive Analytics for the Prediction of Readmission or Mortality

Patients with heart failure face a high risk of mortality and hospital readmission. As with the implementation of predictive analytics to predict heart failure and its risk, this approach can be applied to predicting the hospitalization, readmission, and mortality of patients with heart failure.

Readmission: With a dataset collected by Mount Sinai Hospital in 2014, Shameer et al. used the naïve Bayes model to build a predictive analytics model [31]. Reaching an AUC score of 0.78 with an accuracy of 83.19%, the study was able to predict hospital readmission among heart failure patients. Bat-Erdene et al. developed predictive analytics models involving 52 hospitals from the Korea Acute Myocardial Infarction-National Institute of Health (KAMIR-NIH) registry to predict rehospitalization of patients with acute myocardial infarction (AMI) [32]. This study used a deep learning algorithm to build the predictive model, which resulted in better performance than traditional machine learning models.

Rizinde et al. built machine learning-based predictive analytics models for predicting the risk of hospitalization of patients with heart failure from the medical records of seven hospitals in Rwanda [33]. Their findings showed that their approach was able to predict a high risk of hospitalization with good performance. In a study from the Philippines, Landicho et al. were able to predict the readmission of 322 patients with heart failure from NMMC in Cagayan de Oro City using machine learning-based predictive analytics [34]. Sohrabi et al. built machine learning-based predictive models to predict one- and three-month hospitalization of 230 patients with heart failure collected by medical centers in Iran [35]. The study used a data mining approach to build machine learning-based predictive models, and their models reached up to a 0.73 AUC score.

By utilizing a database from the University of Utah Health Care, AbdelRahman et al. built a machine learning-based predictive model to predict the readmission of patients with chronic heart failure (CHF) [36]. By dividing the dataset used into a three-step approach, this study produced a high-performance predictive analytics model that can be applied in various healthcare areas to improve the effectiveness of patient care. Involving the data of more than 1 million patients from the University of Virginia Clinical Database Repository (CDR), Vedomske et al. built a Random Forest-based predictive analytics model to predict unplanned, all-cause, 30-day readmission of patients with CHF [37]. By processing administrative data, the proposed model reached a 0.8 AUC in their model’s evaluation. Similarly, Hilbert et al. described the implementation of their predictive analytics by demonstrating how their decision tree algorithm produced a transparent analysis of their readmission prediction, resulting in a good AUC score [38].

Zolbanin and Delen proposed a novel data processing approach to extract data from medical records for improved readmission prediction in a sample of heart failure patients [39]. Utilizing data from the Center for Health Systems Innovation (CHSI) at Oklahoma State University (OSU), the proposed model was able to demonstrate competitive advantages in predicting readmission among heart failure patients. Utilizing data from Partners Healthcare System, Golas et al. implemented predictive analytics for the prediction of 30-day hospital readmission of patients with heart failure [40]. By processing structured and unstructured data, the study was able to showcase optimal performance in predicting 30-day readmission through the use of their modified deep learning algorithm.

Two long-term projects, the Tele-HF project and the Gestione Integrata dello Scompenso Cardiaco (GISC) study, built predictive analytics models for readmission [41,42]. The Tele-HF project, involving 1,653 patients, produced a 17.8% improvement in predicting 30-day all-cause readmission. The GISC study compared various machine learning algorithms to predict the hospitalization of patients with heart failure. The study demonstrated various levels of performance in predicting readmission.

Differing from other studies, Sundararaman et al. and Liu et al. utilized open, public data from the MIMIC-III dataset for the predictive analytics of their heart failure readmission prediction [43,44]. Sundararaman et al. employed structured and unstructured data to produce logistic regression-based predictive models. By dividing the dataset into five types based on their proposed iteration, they achieved high accuracy and AUC scores. Attempting a different approach, Liu et al. used only unstructured data (clinical notes) to build their NLP CNN-based predictive models, achieving an accuracy of over 70% for predicting readmission.

To validate predictive analytics models for predicting readmission, Sharma et al. developed predictive analytics models with 12 different machine learning algorithms and compared the performance of each model with the LaCE score, a common method used for predicting patient readmission [45]. They demonstrated that most machine learning-based predictive analytics models provided better performance in predicting readmission than did the LaCE score. Shams et al. compared their proposed approach, the Potentially Avoidable Readmission (PAR) approach, with other methods, such as 3M of the Centers for Medicare and Medicaid Services (CMS) and Potentially Preventable Readmission (PPR) [46]. The study showed efficacy in avoiding patient readmission within two weeks post-hospital discharge.

Involving expert cardiologists in the feature selection of the heart failure data collected from Sheba Medical Center, Ben-Assuli et al. implemented three approaches to building their predictive models categorized by the feature selection method: machine-based, human expert-based, and collaboration-based [47]. The study generated better performance in predicting the 30-day readmission of patients with heart failure with collaboration-based feature selection, providing a different perspective on developing predictive hospital readmission analytics.

Mortality: Improved prediction of the risk of death for individual patients with heart failure is invaluable to healthcare professionals and help reverse the trend toward high cost. By leveraging clinical data from Geisinger EHR, nearly 27,000 patients with heart failure and 276,819 episodes, Jing et al. constructed machine learning-based predictive models using a split-by-year training scheme from 2013 to 2018. They presented good accuracy in predicting all-cause mortality for this large cohort [48]. In a study done between 2013 and 2019 that involved data from 70 Japanese hospitals that contributed to the Tokushukai Medical Database, Kamio et al. generated machine learning-based predictive models that included data for in-hospital mortality by intensive care unit-admitted patients with acute heart failure [49]. By proposing three types of data: static, time-series, and a combination, the study produced varying prediction performances for different outcomes.

Utilizing the electronic medical records of 5,822 patients collected by the University of California San Diego (UCSD), Adler et al. generated a new mortality risk predictive analytics model they named MARKER-HF [50]. The proposed model was able to predict the mortality risk separately for high- and low-risk groups based on the MARKER-HF score. Similarly, Lagu et al. compared seven different mortality prediction models to predict the inpatient mortality of hospitalized patients with ADHF collected from the HealthFacts data of Cerner Corporation [51]. Although the study did not generate a new predictive model, it highlighted the performance of existing approaches from various datasets and showcased their effectiveness in predicting mortality. In contrast, Panahiazar et al. demonstrated that the Seattle Heart Failure Model (SHFM) could predict mortality using the Mayo Clinic dataset [52]. However, their study also generated an approach to building predictive models that resulted in better performance than the SHFM approach, with improved prediction accuracy.

The use of open, public data, such as that from the UCI repository and Physionet, benefits researchers in building mortality risk predictive analytics models. Without performing pre-processing, machine learning-based predictive analytics models were built that predicted the survival possibility of heart failure patients in the UCI heart failure dataset [53]. Utilizing the PCA method to determine the effectiveness of dimension reduction in prediction performance, Karakuş and Er used various machine learning algorithms with the UCI heart failure dataset and showed an improvement in classification accuracy [54]. However, because the dataset contains an imbalance class, the study did not perform a balancing method, which may have led to biased prediction results. Therefore, Zaman et al. addressed the imbalance class by employing the SMOTE technique. The proposed models performed better than previous studies. However, Newaz et al. claimed that the SMOTE technique might cause information loss and presented a robust Random Forest classifier, BRF, that could handle imbalance problems with fine prediction performance [56].

Two studies employed feature selection methods for the UCI heart failure dataset [57,58]. While Kedia and Bhushan experienced prediction performance reduction after employing recursive feature elimination, Chicco and Jurman showcased improvement by combining machine learning-based and biostatistical-based feature selection, leading to better performance metrics.

Leveraging the MIMIC-III dataset, Li et al. developed an XGBoost-based predictive analytics model for all-cause in-hospital mortality of ICU-admitted patients with heart failure [59]. With data from 1,177 patients, the study predicted mortality with good AUC-ROC and calibration c-statistic test performance. Similarly, Luo et al. predicted in-hospital mortality among ICU-admitted heart failure patients using the MIMIC-III dataset and the XGBoost algorithm [60]. The study incorporated imputation and feature selection techniques, resulting in a better predictive model with better in-hospital mortality prediction performance than Li et al. Using the updated MIMIC-IV dataset, Chen et al. constructed a predictive analytics model for in-hospital all-cause mortality of ICU-admitted patients with heart failure [61]. The study demonstrated excellent performance by building predictive models using 17 important features selected by LASSO regression.

Both Readmission and Mortality

Various studies have implemented predictive analytics to predict hospital readmission and mortality risk for heart failure patients. Awan et al. aimed to develop machine learning-based predictive analytics models for 30-day heart failure readmission or mortality by extracting datasets from the Hospital Morbidity Data Collection and Mortality Database of the Western Australian Data Linkage System [62]. The study, which was based on data from over 200,000 patients, showed good performance compared to the LaCE score. Awan et al. also proposed different types of predictive model-building by leveraging feature selection and dimension reduction techniques [63]. The study showed improved sensitivity and feature efficiency compared to their previous approach. Similarly, through the use of a core data system, Sarijaloo et al. extracted datasets from EPIC EHR and the McKesson Change ECG Reporting System to build machine learning-based models for the prediction of 90-day acute heart failure readmission or all-cause mortality [64]. By assessing their models with the AUC and 95% CI, the study generated predictive analytics to identify high-risk heart failure patients for either readmission or mortality.

Using a dataset from Shanxi Province, China, Tian et al. generated predictive analytics models to predict death, readmission, and Major Adverse Cardiovascular Events (MACEs) for heart failure patients [65]. The proposed models were able to calculate the risk of death, readmission, and MACEs hospital outpatients with heart failure. Similarly, using electronic health records (EHR) data from the First Affiliated Hospital of Dalian Medical University, China, Lv et al. developed predictive analytics models to predict one-year in-hospital mortality, the use of positive inotropic agents, and one-year all-cause readmission, resulting in good discrimination and predictive performances [66].

The GWTH-HF in-hospital program derived and validated risk-prediction tools from a large nationwide registry [67]. Utilizing the Center for Medicare and Medicaid Services (CMS) tool for predicting mortality and rehospitalization, the study claimed improved mortality and rehospitalization prediction, demonstrating fair discriminative capacity in predicting rehospitalization.

Unlike previously discussed studies, Zhao et al. processed the TOPCAT trial dataset to predict mortality and readmission risk among HFmrEF patients [68]. Their models showed promising prediction performance. Similarly, Beecy et al. used the dataset from the CLEVER-HEART study dataset to build machine learning-based predictive analytics models for predicting 30-day unplanned readmission and all-cause mortality [69]. The proposed models outperformed the HOSPITAL score, indicating superior predictive capabilities.

Discussion

Machine Learning Algorithms for Building Predictive Models

**Figure 2:**
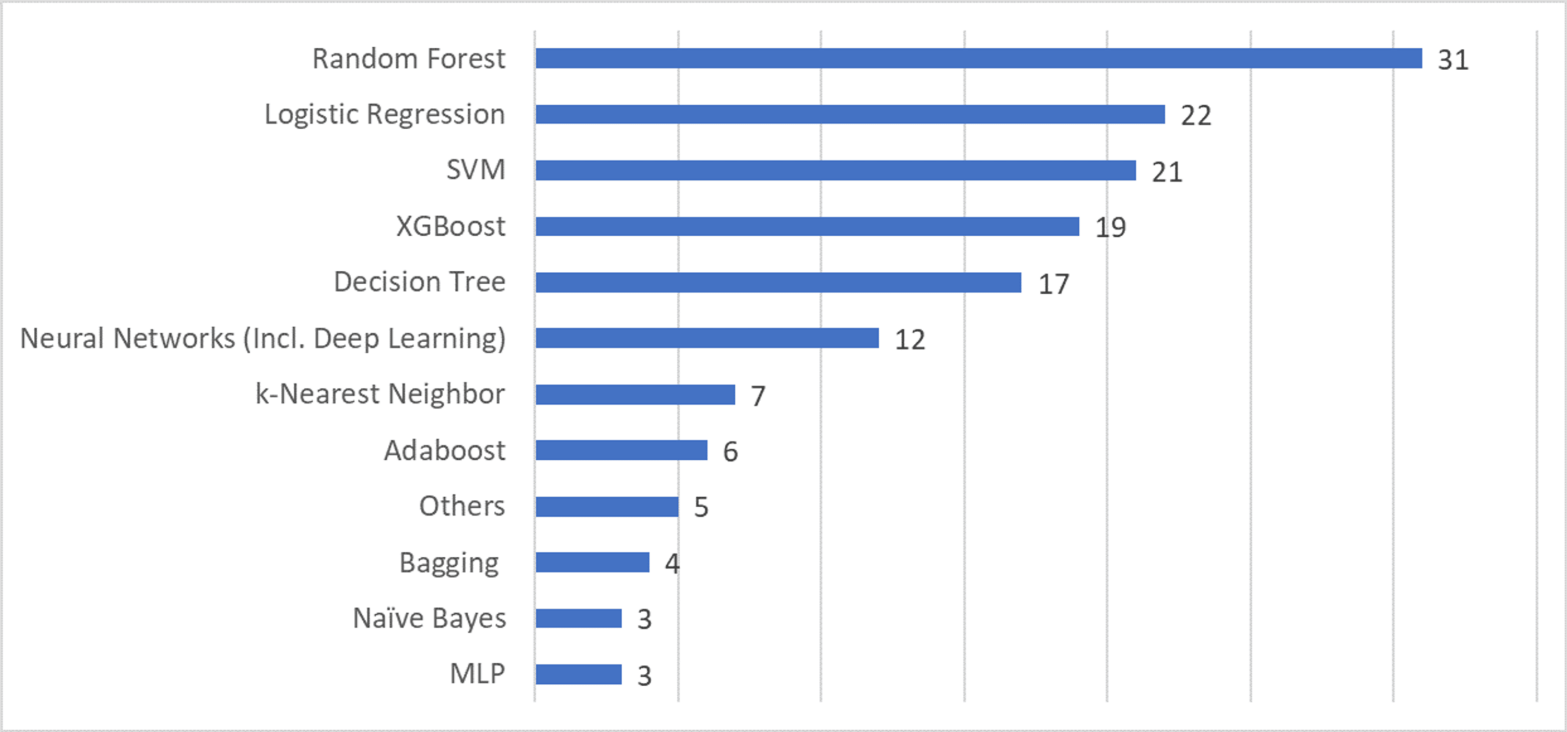
Machine learning algorithms used in selected research articles SVM:Support Vector Machine; XGBoost:eXtreme Gradient Boosting; MLP:Multilayer Perceptron

Machine learning algorithms are essential to building and developing predictive analytics models. The objective of implementing machine learning algorithms is to make the decision-making of predictive analytics more accurate in predicting specific diseases. The selected papers in this literature review present diverse machine-learning algorithms that can be used to predict heart failure, readmission, and patient mortality. As shown in Figure 2, the Random Forest algorithm appeared in 31 articles. It was useful for predicting heart failure, readmission, and mortality. Notably, two studies demonstrated the effectiveness of a Random Forest algorithm when applied to the EHR-based dataset [37,56]. However, an extensive examination of its performance across diverse healthcare datasets is required to measure its trustworthiness in clinical practice. Guidi et al. showed that although the Random Forest algorithm provided the best accuracy in prediction, it provided a less understandable model compared to other algorithms [5]. The implications of this for clinical acceptance need to be carefully considered.

Logistic regression, a traditional machine learning and statistics approach, also improves the robustness of predictive analytics models. Sundararaman et al. indicated a deeper analysis of its predictive potential and its appropriateness for complex clinical settings [43]. Although this algorithm provides simplicity and interpretability, compared to ensemble learning like XGBoost, logistic regression produces lower predictive performance [61].

SVM and Decision Tree (DT) have demonstrated effectiveness in heart failure prediction [34,53]. SVM is known to be an effective nonparametric classification tool, especially for high-dimensional data. However, its complexity results in a lack of interpretability and difficulty in evaluating feature importance [70]. Unlike SVM, DT provides understandable information for identifying risk factors [38]. Nonetheless, a more thorough examination of their results across diverse datasets and potential biases will improve the overall critical discourse. Furthermore, a more comprehensive evaluation is required to confirm their effectiveness in practical applications.

When compared to statistical approach-based predictive models, XGBoost yields superior predictive performance [48]. This algorithm can reduce the likelihood of overfitting when attempting to predict heart failure [60]. However, the intricacy of XGBoost can make interpretation difficult, requiring different approaches for experts looking for clear insights into the decision-making process [14]. Despite its potency, XGBoost has a complexity that provides challenges for real-world deployment and interpretability, which may limit its practical use.

Various selected studies have used neural networks and deep learning to build their predictive analytics. Deep learning algorithms would produce far better performance than conventional machine learning algorithms [32,64]. However, they require massive data, which risks overfitting to narrow datasets [44]. Furthermore, it can be challenging to interpret them due to their “black box” nature [40]. Therefore, their applicability may be limited in real-world clinical settings.

Five of the studies in this literature review used algorithms that were different from those previously mentioned. AbdelRahman et al. used a voting feature classifier model in building their predictive analytics [36]. Lagu et al. modified existing classifier models to predict heart failure mortality [51]. Nagrecha et al. introduced a novel perspective on predicting heart failure based on the unseen disease history of patients by using a directed acyclic graph-based model [9]. Krittayaphong et al. used the Cox Proportional Hazard model to predict heart failure [10]. Eapen et al. used the CMS approach to predict the rehospitalization and mortality of heart failure patients [67]. These five studies highlight the versatility of predictive analytics and demonstrate how various methodologies may predict heart failure risk. Various approaches for building mortality and readmission predictive analytics models, such as CMS, LACE, 3M PPR, and HOSPITAL, are worth considering and evaluating.

Data Pre-processing Implementation in Building Predictive Models

In predictive analytics, obtaining an ideal dataset is a process that allows the predictive models to achieve satisfactory performance in predicting heart failure risk. As a crucial part of building a predictive model, the pre-processing step leads to much better results predicting heart failure than models without pre-processing. Most selected studies implemented a pre-processing stage. Their results showed differences and improvements compared to studies without pre-processing. Table 2 shows the studies that used pre-processing techniques.

**Table 2:** Pre-processing techniques used in selected articles Data cleaning includes removing missing values and performing imputation techniques. Data transformation includes normalization, standardization, and feature selection. Abbreviations: PCA:Principal Component Analysis; SMOTE:Synthetic Minority Over-sampling Technique; ADASYN:Adaptive Synthetic

Pre-processing Step	Article	Frequent method
Data Cleaning	[7,13,19,20,23,26,28,32-36,39-42,45-47,54,55,60,64-66,68,69]	Mean imputation, predictive mean matching, median imputation, random forest imputation, kNN imputation, XGBoost imputation, and missForest
Data Transformation	[13,14,22-26,30,31,33,34,36,39,41-43,47,54,55,58,60,62-65,69]	Recursive feature elimination, SelectKBest, Chi-Square, Pearson's correlation, KS-Test, T-Test
Data Reduction	[47,50,54]	PCA
Data Balancing	[13,14,21-23,32,33,39,43,55,62,65,66]	SMOTE, Under-sampling, Over-sampling, ADASYN

Mean imputation frequently appears in various studies, while the machine learning-based imputation technique obtained better results than statistical-based imputation. Although removing missing values by employing imputation methods is commonplace, a more critical evaluation is necessary to determine their impact on predictive model performance [39].

Only a few of the selected studies used normalization and standardization. However, feature selection appears in many selected studies. Various studies used machine learning-based feature selection in post-processing to describe and present the most important features that influenced their prediction [41]. Although most studies did not compare feature selection methods, comparing them will allow the determination of optimal techniques for use in the pre-processing stage [63]. Furthermore, a comprehensive evaluation is essential to comprehend whether feature selection genuinely improves prediction performance or introduces potential biases. Feature selection risks optimizing models to the specifics of training data rather than generalizable patterns.

PCA was the most frequently used data reduction technique in the selected articles. Nevertheless, a more in-depth analysis is required to understand the implication of dimensionality reduction in preserving essential information. Data balancing was done in studies with imbalanced classes to obtain the ideal dataset. The most used data balancing technique was the SMOTE method. However, a more comprehensive evaluation is needed to determine its effectiveness across different datasets and potential consequences, such as information loss.

Dataset Specification Used in Building Predictive Models

Understanding the dataset specification is essential to building the ideal and optimal predictive analytics models for heart failure prediction with satisfactory performance. Demographic data, prominently age, and sex, frequently appear in selected studies and are used in their predictive analytics building. Ben-Assuli et al. reported that sex and age; expert-recommended demographical data; were the features most closely related to the risk of heart failure [47]. Two studies found age to be the leading feature for predicting 30-day readmission or death [62,63]. These studies give evidence of the importance of the use of demographical data. Through the use of the UCI database involving the feature selection technique, Senan et al. showed that sex and age impacted heart failure in patients [23]. While demographic data is undoubtedly crucial, there is a need for a more nuanced exploration of how each demographic variable contributes to prediction performance. Furthermore, considering factors like lifestyle or comorbidities, a deep analysis could improve prediction performance.

Similarly, the physical and clinical values were essential to building an effective predictive analytics model for heart failure prediction [41]. Physical and clinical values such as weight, diastolic and systolic blood pressure, and heart rate are patient data that clinicians routinely collect. Various studies indicate the importance of physical and clinical values in improving the prediction performance of heart failure predictive analytics models [7,13,41]. Although these values have been shown to be indicators of heart conditions, a more profound exploration is needed to understand the specific relationship between these values and prediction outcomes.

**Figure 3:**
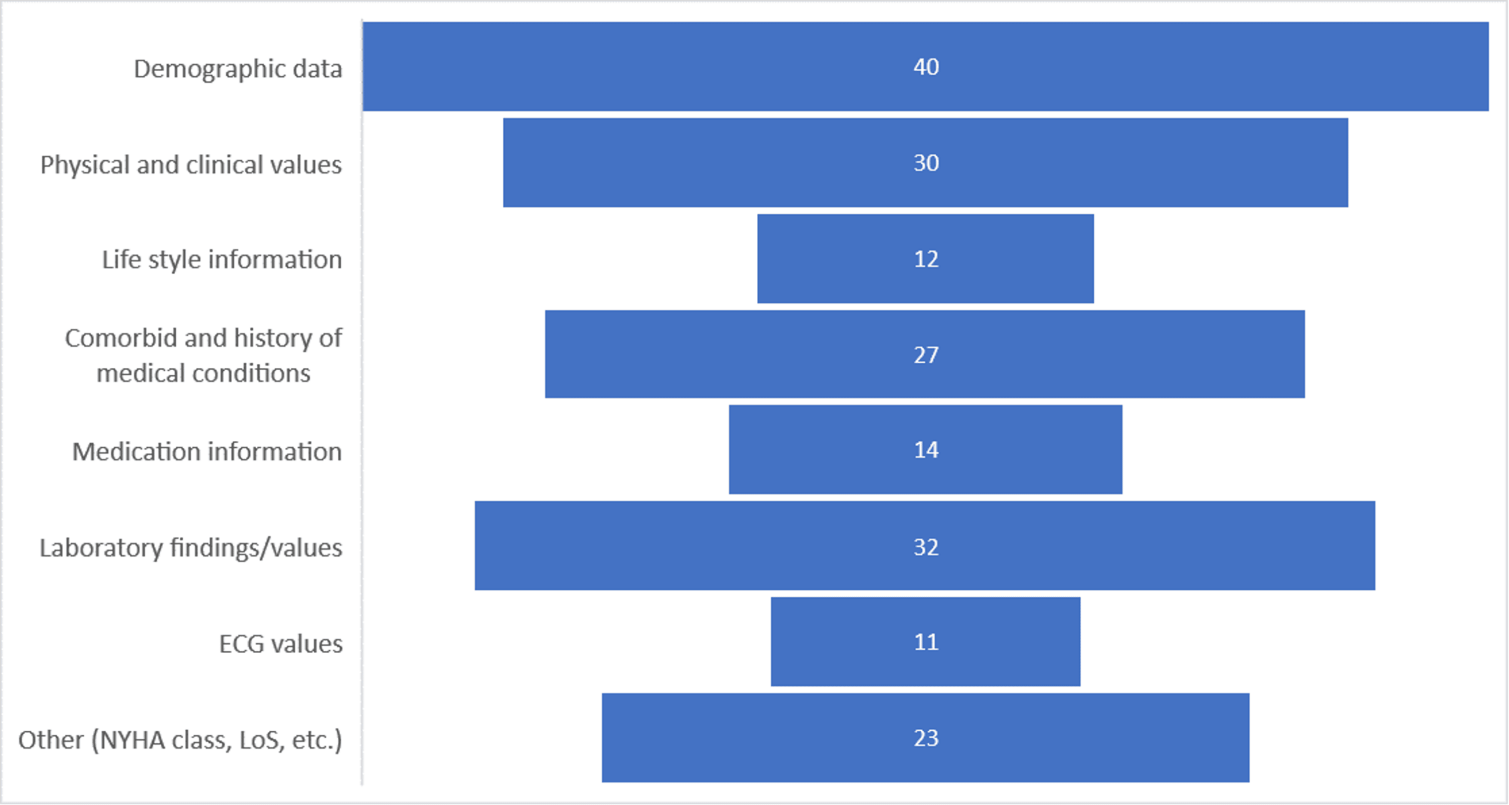
Data categories reported in the selected papers NYHA:New York Heart Association; LoS:Length of Stay

Lifestyle variables, such as smoking and alcohol consumption, introduce a layer of complexity that necessitates a holistic understanding of patient health [6,13]. However, these studies lack a comprehensive investigation of how lifestyle variables contribute to heart failure prediction. In various studies, comorbidities have been essential to building predictive models for the prediction of heart failure or readmission. Panahiazar et al. showed that including comorbidity data improved prediction performance compared to models without comorbidity data [52]. Nevertheless, a deeper exploration of the specific comorbid conditions and their varying impacts on heart failure prediction is warranted. Figure 3 summarizes the data categories described in the selected papers.

According to Figure 3, various studies used data like medication information and ECG values in building their models [29,64]. However, a few studies used unstructured data, such as doctor's notes. By processing patient data, such as patient descriptions, clinical notes, discharge summaries, diagnoses and procedures, and admission events, Sundararaman et al. were able to generate well-performance predictive models [43]. Processing unstructured data to build a heart failure predictive model is suitable for in-house applications like telemedicine, where the clinician collects patient data by phone [41]. Nevertheless, integrating between unstructured and structured variables could unlock richer insights into heart failure risk prediction [40].

Publication by Year

**Figure 4:**
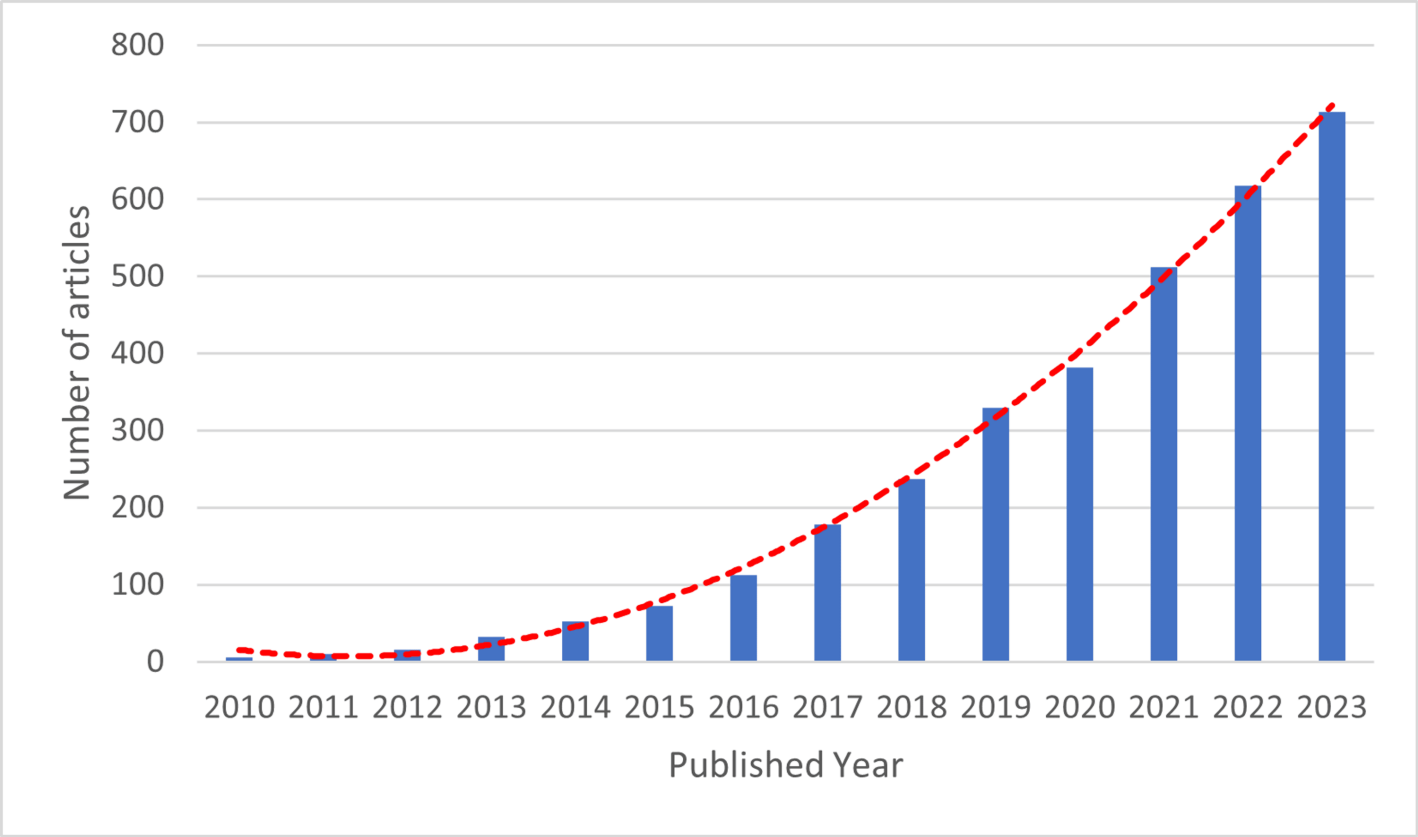
Publication by year based on refined-search results

Although our search was initially set for publications between 2000 and 2023, our refined search mainly generated papers from 2010, as shown in Figure 4.

**Figure 5:**
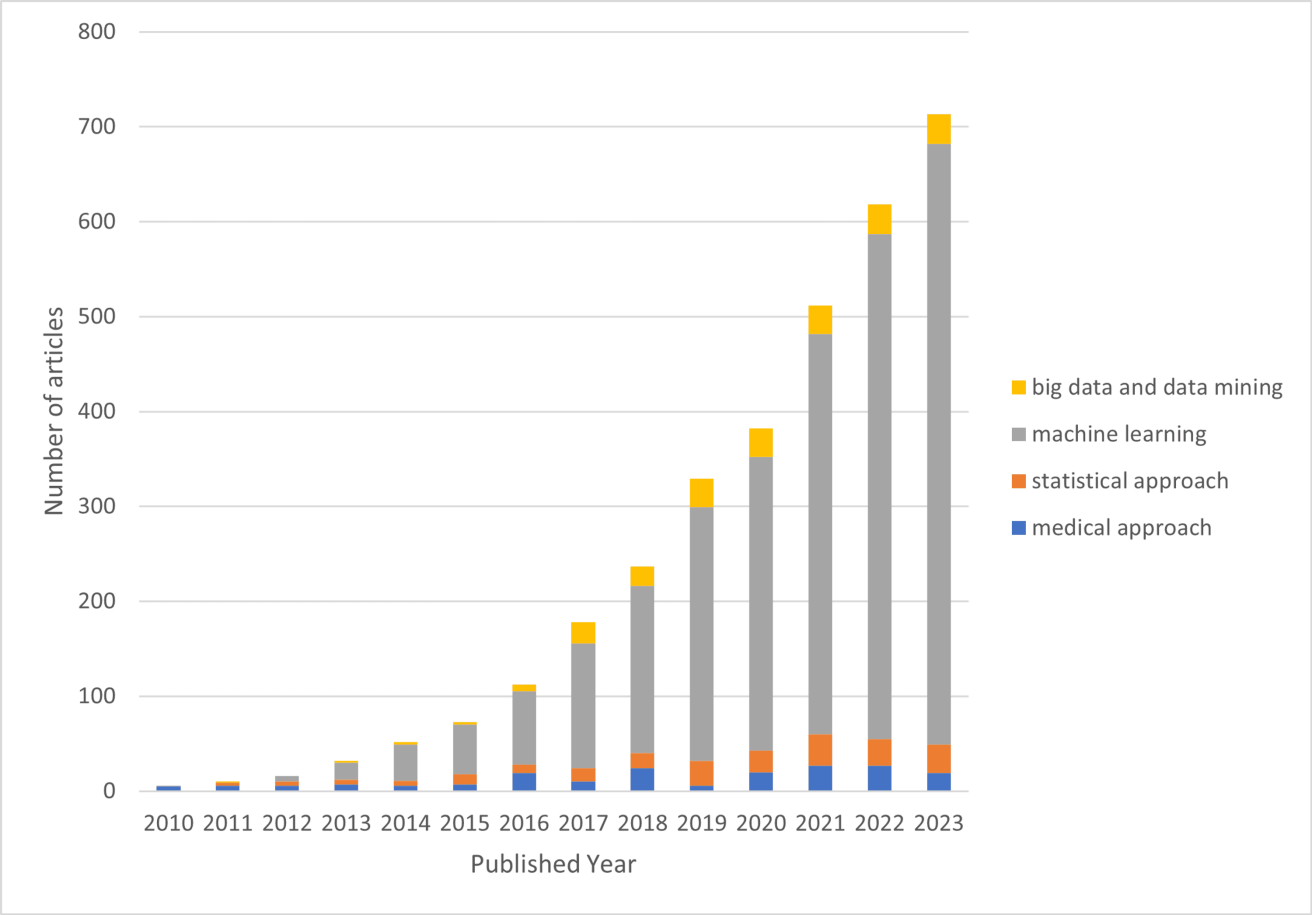
Methodology used in the refined search papers The medical approach, commonly used by medical field experts like doctors and clinicians in presenting their predictive model by using medical or clinical approaches like physical examination and laboratory tests. The statistical approach or statistical analysis approach including Cox hazard proportional approach. The machine learning approach, the artificial intelligence-based approach. The big data and data mining approach, where researchers use various big data applications or software in building predictive models.

We broke down the publications into four well-known methodologies for building predictive models, as presented in Figure 5. As can be seen in the graph, researchers first utilized the machine learning approach in 2012. Its use has increased over time and peaked in 2023 when about 633 studies utilized machine learning to build a predictive model. This approach is currently widespread among researchers, especially for developing predictive analytics models.

Limitation of the study

This review has various limitations, including a restricted search strategy and databases, a lack of dual independent screening and data extraction, and the inability to synthesize results quantitatively across various studies. In the study selection, it is important to acknowledge the subjectivity in the process, which may have biased the results. Although the selected studies utilized datasets from various countries, the transferability of predictive models across diverse populations and settings remains unclear. Future research should encompass quality appraisal, bias assessment, and meta-analysis to derive definitive conclusions about the role and effectiveness of predictive analytics in improving heart failure prognosis. Despite these limitations, this review delivers a foundation for progress toward higher-quality evidence synthesis in this field.

## Conclusions

Predictive analytics and machine learning techniques have demonstrated promising potential for improving early diagnosis of heart failure, stratification of readmission risk, and prediction of mortality for heart failure patients. Various machine learning algorithms like random forest, logistic regression, neural networks, and XGBoost have been applied to structured data from electronic health records and unstructured clinical notes. Appropriate data preprocessing through imputation, feature selection, and the handling of class imbalance have emerged as crucial for developing high-performing predictive models. While the reviewed studies highlight the rising research interest and show initial success, the authors would like to emphasize the need for more rigorous evaluation, head-to-head benchmarking, and quality synthesis to derive more robust evidence that supports the clinical adoption of these predictive analytics approaches for improving heart failure outcomes.
